# A clinical 3D pointing test differentiates spatial memory deficits in dementia and bilateral vestibular failure

**DOI:** 10.1186/s12883-024-03569-4

**Published:** 2024-02-23

**Authors:** J. Gerb, T. Brandt, M. Dieterich

**Affiliations:** 1 Department of Neurology, University Hospital, Ludwig-Maximilians-University, Munich, Germany; 2 Graduate School of Systemic Neuroscience, Ludwig-Maximilians-University, Munich, Germany; 3 German Center for Vertigo and Balance Disorders, University Hospital, Ludwig-Maximilians-University, Munich, Germany; 4 Munich Cluster for Systems Neurology (SyNergy), Munich, Germany

**Keywords:** Spatial memory, Spatial orientation, Pointing task, Bedside test, Dementia, Bilateral vestibulopathy

## Abstract

**Background:**

Deficits in spatial memory, orientation, and navigation are often neglected early signs of cognitive impairment or loss of vestibular function. Real-world navigation tests require complex setups. In contrast, simple pointing at targets in a three-dimensional environment is a basic sensorimotor ability which provides an alternative measure of spatial orientation and memory at bedside. The aim of this study was to test the reliability of a previously established 3D-Real-World Pointing Test (3D-RWPT) in patients with cognitive impairment due to different neurodegenerative disorders, bilateral vestibulopathy, or a combination of both compared to healthy participants.

**Methods:**

The 3D-RWPT was performed using a static array of targets in front of the seated participant before and, as a transformation task, after a 90-degree body rotation around the yaw-axis. Three groups of patients were enrolled: (1) chronic bilateral vestibulopathy (BVP) with normal cognition (*n* = 32), (2) cognitive impairment with normal vestibular function (*n* = 28), and (3) combined BVP and cognitive impairment (*n* = 9). The control group consisted of age-matched participants (HP) without cognitive and vestibular deficits (*n* = 67). Analyses focused on paradigm-specific mean angular deviation of pointing in the azimuth (horizontal) and polar (vertical) spatial planes, of the preferred pointing strategy (egocentric or allocentric), and the resulting shape configuration of the pointing array relative to the stimulus array. Statistical analysis was performed using age-corrected ANCOVA-testing with Bonferroni correction and correlation analysis using Spearman’s rho.

**Results:**

Patients with cognitive impairment employed more egocentric pointing strategies while patients with BVP but normal cognition and HP used more world-based solutions (pBonf 5.78 × 10-3**). Differences in pointing accuracy were only found in the azimuth plane, unveiling unique patterns where patients with cognitive impairment showed decreased accuracy in the transformation tasks of the 3D-RWPT (pBonf < 0.001***) while patients with BVP struggled in the post-rotation tasks (pBonf < 0.001***). Overall azimuth pointing performance was still adequate in some patients with BVP but significantly decreased when combined with a cognitive deficit.

**Conclusion:**

The 3D-RWPT provides a simple and fast measure of spatial orientation and memory. Cognitive impairment often led to a shift from world-based allocentric pointing strategy to an egocentric performance with less azimuth accuracy compared to age-matched controls. This supports the view that cognitive deficits hinder the mental buildup of the stimulus pattern represented as a geometrical form. Vestibular hypofunction negatively affected spatial memory and pointing performance in the azimuth plane. The most severe spatial impairments (angular deviation, figure frame configuration) were found in patients with combined cognitive and vestibular deficits.

**Supplementary Information:**

The online version contains supplementary material available at 10.1186/s12883-024-03569-4.

## Introduction

Deficits in spatial orientation and spatial memory are early signs of cognitive decline, e.g., in Alzheimer’s disease (AD, [1, 2]) and other forms of dementia [3, 4]. Since they often are the first cognitive symptoms with a long time span of preclinical stages of dementia [5] in which therapeutical interventions might be possible, the importance of testing spatial orientation and memory becomes clinically relevant. Such a test could be used as a neuropsychological biomarker for early diagnosis and disease progression [6, 7]. However, there is still a need for an easy-to-use, 3D real-world, clinical bedside test of these orientational abilities which ideally allows for the distinction between perceptual deficits (e.g., bilateral vestibular loss) and dysfunction of cortical processing (different forms of dementia).

Apart from degenerative and vascular cognitive neurological disorders, chronic peripheral bilateral vestibulopathy (BVP) also significantly affects spatial orientation and spatial memory associated with hippocampal atrophy [8–11]. The latter deficits have only been proven in recent years. These findings prompted us to test this group of patients in whom the disorder affects primarily the peripheral organ rather than cortical mechanisms as in various forms of dementia. There is a clinical need to differentiate the specific deficits of the three conditions, a general cognitive decline, a sensory loss of vestibular function, and the combination of both which is not rare in the elderly [12–14], in order to provide early therapeutical options (e.g., cognitive training, vestibular rehabilitation). There are a number of tests available for spatial abilities such as questionnaires, pen-and-paper-tests, digital navigation in virtual environments, real-life navigation tasks, or tasks with stationary subject-world interaction [15–17]. Most of these tests require time consuming complex setups or their measures are restricted to 2D rather than 3D orientation. In general, clinical assessment of spatial orientation and memory should be based on actual performance as in the 2D pen-and-paper test [15] or a 3D smartphone-based finger-arm-pointing test [18, 19], since the patient’s history of self-estimated navigation ability might reflect subjective misjudgments [20–22]. The here used 3D Real-World Pointing Test (3D-RWPT) requires the participant to update their reference frame of the environment after horizontal whole-body rotations ( ≙ yaw-axis rotation) in order to still be able to correctly interact with static real-world targets, and gives additional information on polar (vertical) as compared to azimuth (horizontal) spatial performance, which may remain undetected in 2D-pen-and-paper tests [18].

For the 3D-pointing paradigm (3D-RWPT [18])] using a previously optimized smartphone-based device [19, 23], we additionally introduced figure frame analysis, which conceptually allows for a separation between impaired *primary figure frame* (on the perception level, e.g., due to decreased visual acuity), *secondary figure frame* (on the mental processing level, e.g., in neurodegeneration), and *tertiary figure frame* (on the output level, e.g., motor impairment) [24]. This data analysis method was inspired by concepts from cognitive psychology (i.e., stimulus perception, integration/interpretation, behavior modification; [25]). In the current study, we used the 3D-RWPT in three groups of patients: degenerative cognitive impairment due to heterogeneous neurodegenerative conditions, bilateral vestibulopathy (BVP), and a combination of both conditions in comparison to a group of age-matched participants without cognitive and vestibular deficits.

The major goals of this study were: to disclose differences in the accuracy of spatial pointing abilities before and after body rotation (spatial transformation and post-transformation of the spatial target array), the preferred use of either egocentric or world-based allocentric pointing strategies, and the consequences of the combination of both diseases. Our hypotheses were that (a) patients with BVP would show higher deviations in the post-rotation task due to the lack of labyrinthine function; (b) patients with cognitive decline and associated visuospatial deficits would show higher deviations in the transformation tasks due to the complexity of the required mental transformation steps to update the spatial map of the environment; (c) the overlap group of BVP and cognitive decline would show the highest deviations; and (d) a shift towards retinotopic/egocentric spatial encoding strategies would be observable in patients with cognitive impairment. We selected patients with vestibular or cognitive deficits in order to compare the effects of a peripheral sensory (vestibular) and a central processing deficit (cognitive) for dynamic spatial orientation. The analyses focused on paradigm-specific mean angular deviation of pointing in the azimuth (horizontal) and polar (vertical) planes, the preferred pointing strategy, and the resulting shape configuration of the pointing results relative to the stimulus array.

## Methods

### Patients

The patients included were examined in our tertiary interdisciplinary center for vertigo and balance disorders (German Center for Vertigo and Balance Disorders) and the Department of Neurology, Ludwig-Maximilians-University, Munich, Germany, between September 2020 and May 2023. Forty-one patients (17 females, mean age 59.95 ± 14.66 years) with chronic bilateral vestibulopathy (BVP) as defined by the Consensus Committee of the Bárány Society [26] were selected. Of these patients, 9 (6 females) had a relevant cognitive deficit, resulting in two subgroups, first BVP with normal cognition (*n* = 32, mean age 58.94 ± 15.40 years), and second BVP with cognitive deficits (*n* = 9, mean age 63.56 ± 11.72 years). The etiology of the BVP was deficiencies due to ototoxic drugs (*n* = 7), bilateral Menière’s disease (*n* = 3), autoimmune disorders (*n* = 3), consequence of ENT-surgery (*n* = 3) with the remainder being idiopathic BVP (*n* = 25). Furthermore, 67 participants (37 females, mean age 48.42 ± 18.17 years) without peripheral-vestibular dysfunction and normal cognition as well as 28 patients without peripheral-vestibular dysfunction but cognitive impairment (14 females, mean age 72.36 ± 7.87 years) were included. All patients and participants underwent the Montreal Cognitive Assessment test (MoCA, [27]) or comparable test batteries (e.g., Mini Mental Test) as a first cognitive screening step. In patients with severe cognitive deficits (MoCA – Score below 24) extensive neuropsychological testing was recommended and partially performed in-house, e.g., with the CERAD (Consortium to Establish a Registry for Alzheimer’s Disease) - test battery. Cognitive impairment was defined by scores equal to or lower than 25 points in the MoCA or similar test. Sufficient hearing status was ensured. Borderline test results (MoCA-Scores 24 and 25, [28]) were correlated with additional detailed neuropsychological testing, if available, and a clinical neurocognitive assessment. Final diagnoses according to the respective diagnostic criteria were vascular encephalopathy (*n* = 12), idiopathic Parkinson syndrome (IPS, *n* = 3), frontotemporal dementia (FTD, *n* = 2), Alzheimer’s disease (AD, *n* = 2), progressive supranuclear palsy (PSP, *n* = 1), normal pressure hydrocephalus (NPH, *n* = 1), and mixed dementia with epilepsy (*n* = 1). Eight patients, all showing clear clinical characteristics of neurodegenerative processes, have not received a final diagnosis yet, while six patients were lost to follow-up. Patients with severe cognitive impairment would have been excluded if they were unable to understand the instructions. This was not the case in the enrolled patients. Further exclusion criteria were relevant impairments of arm function due to, e.g., cerebellar ataxia, tremor, paresis, or orthopedic disorders as well as uncorrected hearing loss.

The data protection clearance and Institutional Review Board of the Ludwig-Maximilians-University, Munich, Germany approved the study (no. 094 − 10), and all patients gave informed consent. The study was performed in accordance with the ethical standards laid down in the 1964 Declaration of Helsinki and its later amendments.

### Neurological and neuro-otological examination

Clinical testing included a neurological and neuro-orthoptic assessment (spontaneous and head-shaking nystagmus, ocular motor examination, fundus photography and adjustment of the subjective visual vertical (SVV) in order to assess vestibular deficits and acute vestibular tone imbalances [29, 30]). Further, bithermal caloric testing using warm (44 °C) water irrigation in the right (WR) and left (WL) as well as cold (30 °C) water irrigation in the right (CR) and left (CL) external ear were performed to measure the function of the horizontal semicircular canals in the low-frequency range of the vestibulo-ocular reflex [31], and standardized vHIT measurements of the semicircular function in the high-frequency range using the EyeSeeCamHIT® system (EyeSeeTec, Munich, Germany) [32]. All participants were tested for handedness by using the Edinburgh Handedness Inventory [33].

### Psychometric testing

The psychometric questionnaire battery consisted of (a) the Santa Barbara Sense of Direction Scale (SBSODS, [15]), (b) the Patient Health Questionnaire subsection 9 (PHQ-9, [34]), and (c) the Montreal Cognitive Assessment (MoCA, [27, 28]; scores were corrected for education level and cross-referenced with clinical cognitive assessment by a medical doctor). Some questionnaires not yet available in a German version had been translated using the cross-cultural adaptation process [35] for prior studies. SBSODS, PHQ-9 and EHI were filled out by the participants themselves without supervision or time constraints, while the MoCA-test was performed in a standardized fashion either by a medical doctor or a registered study nurse. For further analysis, the SBSODS was divided into questions with an emotional element (e.g., “I don’t enjoy giving directions.”, items 6, 7, 8, 10, 13), questions on absolute self-assessed function (e.g., “I am very good at judging distances.”, items 1, 2, 3, 4, 9, 10, 14), and questions where the score does not necessarily indicate higher or lower orientational performance but possibly individual preferences (e.g., “I tend to think of my environment in terms of cardinal directions (N, S, E, W).”, items 5, 12, 15).

### Pointing test procedure (3D-real-world pointing test)

**Fig. 1:**
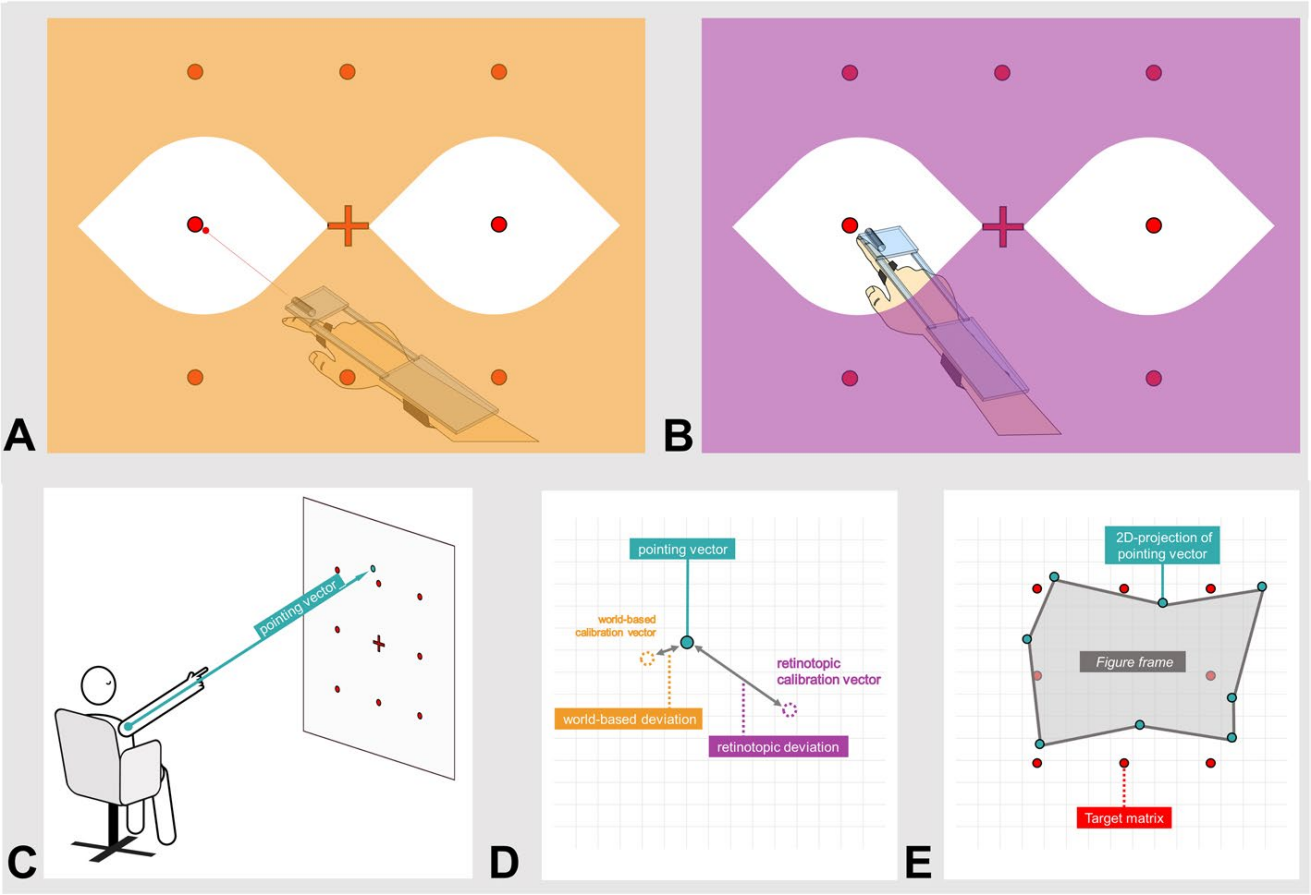
Depiction of the pointing task data processing steps. **A** World-based allocentric calibration is performed with open eyes and a laser pointer. Here, the subject aligns the real-world pointing trajectory (= laser dot) with the real-world target. **B** Retinotopic egocentric calibration is performed with open eyes. Here, the subject aligns their retinotopic visual representation of the extended hand/finger with the real-world target. **C** The participant is given instructions to point towards the different targets with their eyes closed (petrol vector). **D** Symbolic cartesian representation of angular deviation calculation in spherical coordinates. Based on each pointing vector (petrol dot), azimuth (φ) and polar (ϑ) deviations from both world-based (orange) and retinotopic (purple) calibration were calculated and averaged over each pointing paradigm. **E** The resulting pointing vectors can be holistically assessed when taking the resulting figure frame, i.e., shape configuration, into account, allowing for morphometrical analysis

**Fig. 2:**
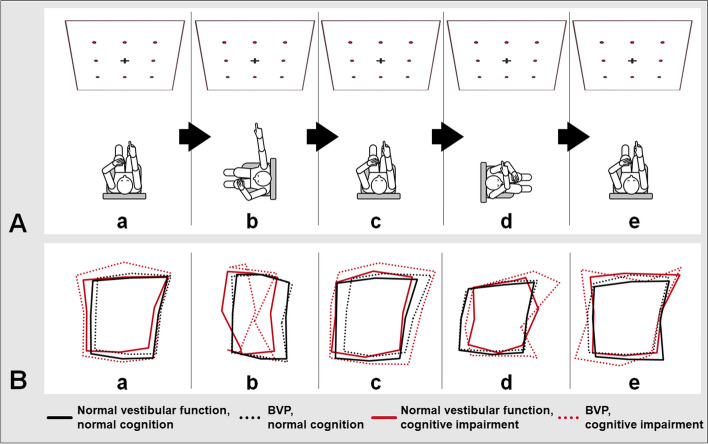
Depiction of the 3D-RWPT paradigms and resulting figure frames. **A** The subject was seated on a swivel chair in a standardized centered position in front of a white wall with a nine-point matrix (target arrangement) marked on it. The pointing device was calibrated to each point in a randomized order. Afterwards, the subject was asked to point to each target with their eyes closed in this initial position (a), following a passive 90° rotation to the non-dominant-hand side performed with visual feedback available during the rotation (b), back in the starting position following a passive rotation without visual feedback during the rotation (c), following a 90° passive rotation to the dominant-hand side with visual feedback available during the rotation (d), and back in the starting position without visual feedback during the rotation (e). **B** When plotting the resulting pointing vectors, the groupwise shape configurations can be analysed (a-e; black solid line figure frames: participants without cognitive or vestibular impairment; black dotted line figure frames: patients with BVP and normal cognition; red solid line figure frames: patients with cognitive impairment and normal peripheral-vestibular function; red dotted line figure frames: patients with cognitive impairment and BVP)

The clinical pointing task was recorded using a smartphone-based (iPhone®, Apple, CA, USA) pointing device, using the built-in 6-axis accelerometer/gyroscope unit (Invensense (TDK), CA, USA), and testing setup from previous work [18, 23], i.e., two calibration and five testing paradigms (Figs. 1 and 2A). The test was administered by one of the authors (JG) or a registered study nurse specifically trained in the correct test execution. Calibration (i.e., pointing to all targets in randomized order with open eyes and visual feedback available) was performed twice: first for the world-based calibration with a laser pointer attached to the device visualizing the real-world pointing vector, and then again for the retinotopic calibration where participants were instructed to visually align each target and their extended index finger. Targets were 20 mm red points on a white wall in a 3 × 3 matrix with 100 cm distance between points. Participants were seated on a swivel chair with their eye level aligned with the center row of dots. An eye-to-wall distance of 192 cm (limited by room size) was chosen to ensure that all targets were located in the near-peripheral field of vision (up to 30° radially outwards, [36]) when participants’ gaze was straight ahead on the central target. In the target matrix, neighboring points were separated by 27.5°, totaling a target angular scope of 55° x 55° in azimuth and polar directions, respectively. For each task, a computerized voice from the device gave a command, e.g., “top left”, and the subjects pointed towards the target with an extended arm. The volume of the commands was adjustable up to 105 dB to ensure sufficient understanding; if necessary, the instructions were repeated by the test supervisor (e.g., when patients were distracted). The measurement was confirmed using a wireless Bluetooth dongle either by the examiner or by the subject themselves. If participants were unable to perform the calibration steps, the experiment was terminated at this stage. After calibration, the subjects were asked to point to the targets in another randomized order (indicated by the device) without visual feedback while facing straight ahead (1), after being passively 90° rotated to their “non-hand-dominant” side (i.e., towards the left side for right-handed participants, towards the right side for left handed participants; hand dominance determined by the EHI) with visual feedback available during rotation and pointing thereafter during eyes closed (2), back in the initial position without visual feedback during rotation (3), after being passively rotated 90° to their “hand-dominant” side with visual feedback available during rotation but eyes closed when pointing (4) and back to the initial target-facing position without visual feedback during rotation (5). Each test was separated by a standardized pause of 30 s signaled in five-second intervals using a notification sound. Participants who showed a relevant egocentric fallback in the rotation tasks (i.e., pointing as if no rotation had taken place, [18]) were documented by the examiner. The average testing time (including participant instructions) took 7 to 10 minutes depending on participant compliance.

### Statistics

After data collection, all data was irreversibly anonymized for data analysis and processed using Microsoft® Excel (Version 2022) and JASP (Version 0.18.0 [37]). For data description, mean values and standard deviation for continuous variables and absolute and relative frequencies for categorical variables were used. We tested statistical inference using Spearman’s rho and performed either independent samples student’s t-test or one-way analysis of variance (ANCOVA) testing for each pointing paradigm when comparing each subgroup (correction for respective covariates depending on subanalysis, post-hoc-testing using 1000 bootstraps, Bonferroni-correction and effect size estimation with partial η^2^, all analyses performed in JASP).

### Pointing task analysis

The pointing vectors from each 3D-RWPT task were used to calculate mean angular deviations in the azimuth (≘horizontal) and polar (≘ vertical) plane relative to the two sets of calibrations, as described in previous studies [18, 23]. We corrected for age, since a shift in navigation strategy can be seen in older age without (known) cognitive decline [38]. The mean absolute deviation between each pointing vector and either world-based or retinotopic calibration vector was computed as a marker of participant performance or ‘accuracy’ with lower mean deviations equalling a higher accuracy (lowest possible value: 0°). The deviation was calculated in azimuth and polar planes for all five tasks and further divided for the initial reproduction task, the transformation tasks (when rotated to dominant and non-dominant side), and the postrotational tasks. Additionally, we calculated ‘directional’ deviations or the ‘precision’ for each paradigm and calibration, i.e., using the non-absolute values. This was necessary in order to separate inaccurate performances with an underlying ‘intact’ mental coding from systematically shifted mental representation and unsystematic pointing performances. Here, a value close to 0° implies an overall intact mental representation (since directional deviations average out), while systematic shifts would result in stable, distinctly positive, or negative deviations. To differentiate pointing performances with an intact mental representation from performances with unsystematic deviations which, due to a stochastic central tendency, might also average out to ~ 0°, one has to furthermore analyse the paradigm-wise standard deviation as a marker for participant consistency (mean azimuth pointing consistency, mAPC, and mean polar pointing consistency, mPPC). Higher values in this metric indicate an unsystematic performance.

Furthermore, by subtracting the mean absolute task-specific deviation calculated with the world-based calibration from the mean absolute deviation calculated with the retinotopic calibration, a metric for the employed pointing strategy was derived. A subject performing the tasks mostly based on a retinotopically coded mental map will show higher deviations from the world-based calibration than from the retinotopic calibration, resulting in a negative value for this metric regardless of overall pointing accuracy (positive values: more world-based coding, negative values: more retinotopic coding, values around zero: no clear preference).

### Figure frame analysis

To further understand groupwise differences, we used a method of creating a 2D projection of the raw pointing vectors as a dimensionless depiction of the underlying mental shape representation of the target array. A detailed description of the figure frame creation steps can be found in [24]. Figure frame analysis is a promising holistic approach to pointing performance analysis, allowing for more complex mathematical analyses. All figure frames were plotted using ImageJ/FIJI [39] while we used the MorphoLibJ-plugin [40] for further morphological analysis, namely rectangularity, perimeter, maximum *feret* diameter (i.e., the maximum distance between two parallel tangential lines), figure area, and offset from the projections’ center.

## Results

Neurotological testing confirmed the peripheral loss of vestibular function in both BVP groups and the normal vestibular function in the other two groups (HC and cognitive impairment; Table 1). The BVP group with and without cognitive impairment had comparable demographics (BVP and normal cognition: 32 patients (11 females), mean age 58.94 ± 15.40 years; BVP and cognitive impairment: 9 patients (6 females), mean age 63.56 ± 11.72 years); the two other subgroups (normal vestibular function with and without cognitive impairment) diverged in their mean age (normal vestibular function + normal cognition: 48.42 ± 18.17 years, normal vestibular function + cognitive deficit: 72.36 ± 7.87 years). Further demographic details and neuro-otological test results can be seen in Table 1.

**Table 1:** Demographic characteristics and results of vestibular testing

	Normal vestibular function, normal cognition	BVP, normal cognition	Normal vestibular function, cognitive deficit	BVP, cognitive deficit
N (of which females)	67 (37)	32 (11)	28 (14)	9 (6)
Mean age in years	48.42 ± 18.17	58.94 ± 15.40	72.36 ± 7.87	63.56 ± 11.72
Mean MOCA score	28.86 ± 1.41	28.12 ± 1.48	22.52 ± 3.14	23.13 ± 3.14
vHIT left side gain (60ms)	1.02 ± 0.20	0.32 ± 0.26	0.94 ± 0.24	0.30 ± 0.18
vHIT right side gain (60ms)	0.99 ± 0.26	0.29 ± 0.20	0.91 ± 0.29	0.35 ± 0.25
mean caloric slow phase velocity (WR)	-19.43 ± 14.36°/s	-1.08 ± 1.50 °/s	-20.80 ± 14.44°/s	-0.90 ± 1.94°/s
mean caloric slow phase velocity (WL)	22.40 ± 14.72°/s	1.77 ± 1.36°/s	24.14 ± 17.50°/s	1.44 ± 1.47°/s
mean caloric slow phase velocity (CR)	14.22 ± 7.23°/s	1.68 ± 1.55°/s	13.68 ± 8.57°/s	2.08 ± 2.26°/s
mean caloric slow phase velocity (CL)	-20.07 ± 9.49°/s	-2.52 ± 4.49°/s	-17.23 ± 8.49°/s	-1.31 ± 1.47°/s

Seven out of all participants (5.15%) were left-handed and performed the test in a mirrored version, i.e., with the device strapped to their left wrist and performing the initial rotation towards the right side instead of the left side, since the first rotation was always towards the non-hand-dominant side. For later data analysis steps, relative directions in regard to hand dominance were used instead of absolute directions such as left or right.

**Fig. 3:**
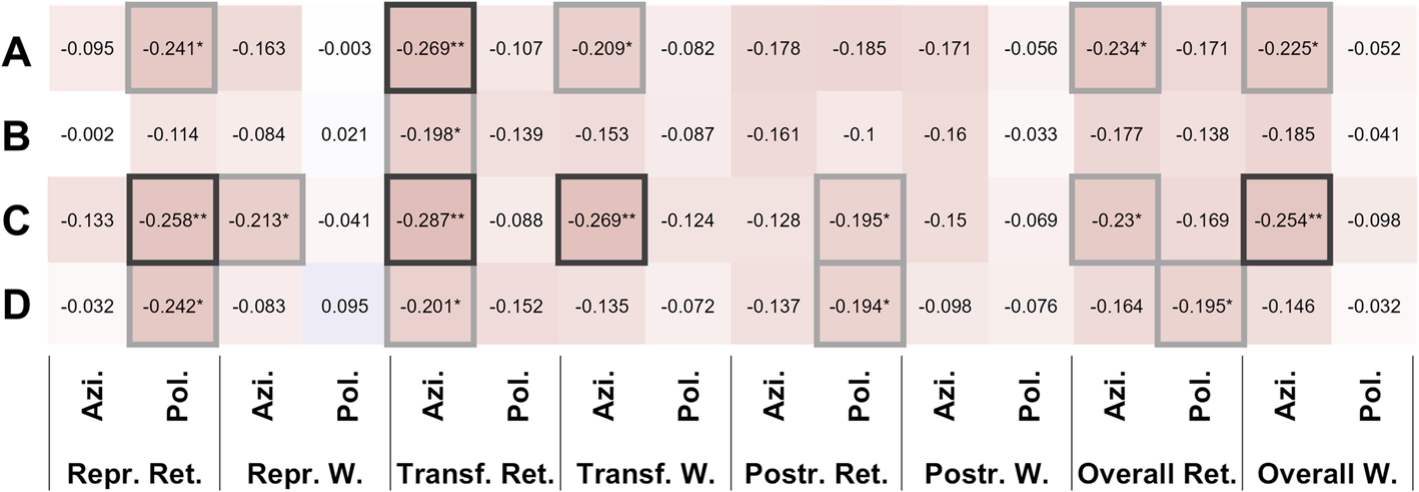
Heatmap of correlation (Spearman’s rho) between self-reported overall orientation skills in the SBSODS (**A**), further subdivided SBSODS subitems **B **items with emotional component; **C **questions on absolute self-assessed function; **D** questions not necessarily indicating orientational performance but rather individual preferences) and pointing deviation in the 3D-RWPT divided by paradigm and calibration (red = negative correlation, blue = positive correlation, darker colors equal stronger correlation). Significant correlations have been marked with boxes (light grey = *p* < 0.05, dark grey = *p* < 0.01)

*Psychometric testing* ruled out relevant group differences in a depression screening questionnaire (PHQ9) with all groups showing similar values and ANCOVA-testing yielding non-significant results (normal peripheral-vestibular function vs. BVP: F(1,118) = 0.33, p 0.57, partial η^2^ = 2.67 × 10-3, post-hoc difference =-0.60, pBonf 0.57; normal cognition vs. cognitive impairment: F(1,118) = 1.71, p 0.19, partial η^2^ = 0.01, post-hoc difference = -1.44, pBonf 0.19). The self-assessment of navigation ability (SBSODS) showed no group differences after correction for participant age, neither in the overall score nor in the subsets (detailed ANCOVA-results in Additional file 1). The correction for participant age was included due to a positive correlation between participant age and the neutral subset of SBSODS-items (Spearman’s rho 0.30, p 1.19 × 10-3**). None of the individual questionnaire items showed significant group differences. Overall and subitem scores partially correlated with pointing performance, especially the functional subitems (i.e., items 1, 2, 3, 4, 9, 10, 14; Fig. 3).

*Mean azimuth deviations (mAD)* from both retinotopic and world-based calibrations were higher in the patients with cognitive impairment and the BVP group, respectively, with the combined patient group (BVP plus cognitive impairment) showing the highest deviations in this “accuracy”-metric (overall mAD retinotopic: normal peripheral-vestibular function + normal cognition 8.49° ± 3.53°, BVP + normal cognition 10.01° ± 4.32°, normal peripheral-vestibular function + cognitive impairment 12.06° ± 5.21°, BVP + cognitive impairment 18.33° ± 9.71°; overall mAD world-based: normal peripheral-vestibular function + normal cognition 8.71° ± 3.23°, BVP + normal cognition 9.63° ± 4.51°, normal peripheral-vestibular function + cognitive impairment 13.89° ± 7.18°, BVP + cognitive impairment 19.60° ± 9.50°; ANCOVA: normal peripheral-vestibular function vs. BVP: pBonf < 0.001*** (retinotopic and world-based), normal cognition vs. cognitive impairment: pBonf < 0.001*** (retinotopic), pBonf 3.75 × 10-3** (world-based), detailed age-corrected ANCOVA-testing in Additional file 1).

Paradigm-wise analysis showed distinct spatial impairment patterns with cognitive impairment causing higher azimuth deviations in the transformation tasks while patients with BVP (but normal cognition) showed testing results almost on the HP-level (overall mAD retinotopic: normal peripheral-vestibular function + normal cognition 10.75° ± 5.38°, BVP + normal cognition 11.71° ± 5.46°, normal peripheral-vestibular function + cognitive impairment 18.15° ± 11.80°, BVP + cognitive impairment 25.99° ± 17.34°; overall mAD world-based: normal peripheral-vestibular function + normal cognition 10.95° ± 5.04°, BVP + normal cognition 10.62° ± 3.81°, normal peripheral-vestibular function + cognitive impairment 19.60° ± 12.69°, BVP + cognitive impairment 27.04° ± 16.68°; ANCOVA: normal peripheral-vestibular function vs. BVP: pBonf 0.02* (retinotopic), pBonf 0.05* (world-based), normal cognition vs. cognitive impairment: pBonf < 0.001*** (retinotopic and world-based), detailed age-corrected ANCOVA-testing in Additional file 1).

Vestibular impairment, however, caused higher azimuth deviations in the post-rotation tasks, where instead patients with cognitive impairment (but normal vestibular function) showed results closer to the normal participants (overall mAD retinotopic: normal peripheral-vestibular function + normal cognition 8.01° ± 3.63°, BVP + normal cognition 10.78° ± 5.53°, normal peripheral-vestibular function + cognitive impairment 9.07° ± 3.79°, BVP + cognitive impairment 17.20° ± 10.47°; overall mAD world-based: normal peripheral-vestibular function + normal cognition 8.20° ± 3.45°, BVP + normal cognition 10.71° ± 6.81°, normal peripheral-vestibular function + cognitive impairment 11.21° ± 6.51°, BVP + cognitive impairment 18.60° ± 10.98°; ANCOVA: normal peripheral-vestibular function vs. BVP: pBonf < 0.001*** (retinotopic and world-based), normal cognition vs. cognitive impairment: pBonf 6.17 × 10-3** (retinotopic), pBonf < 0.001*** (world-based), detailed age-corrected ANCOVA-testing in Additional file 1).

**Fig. 4:**
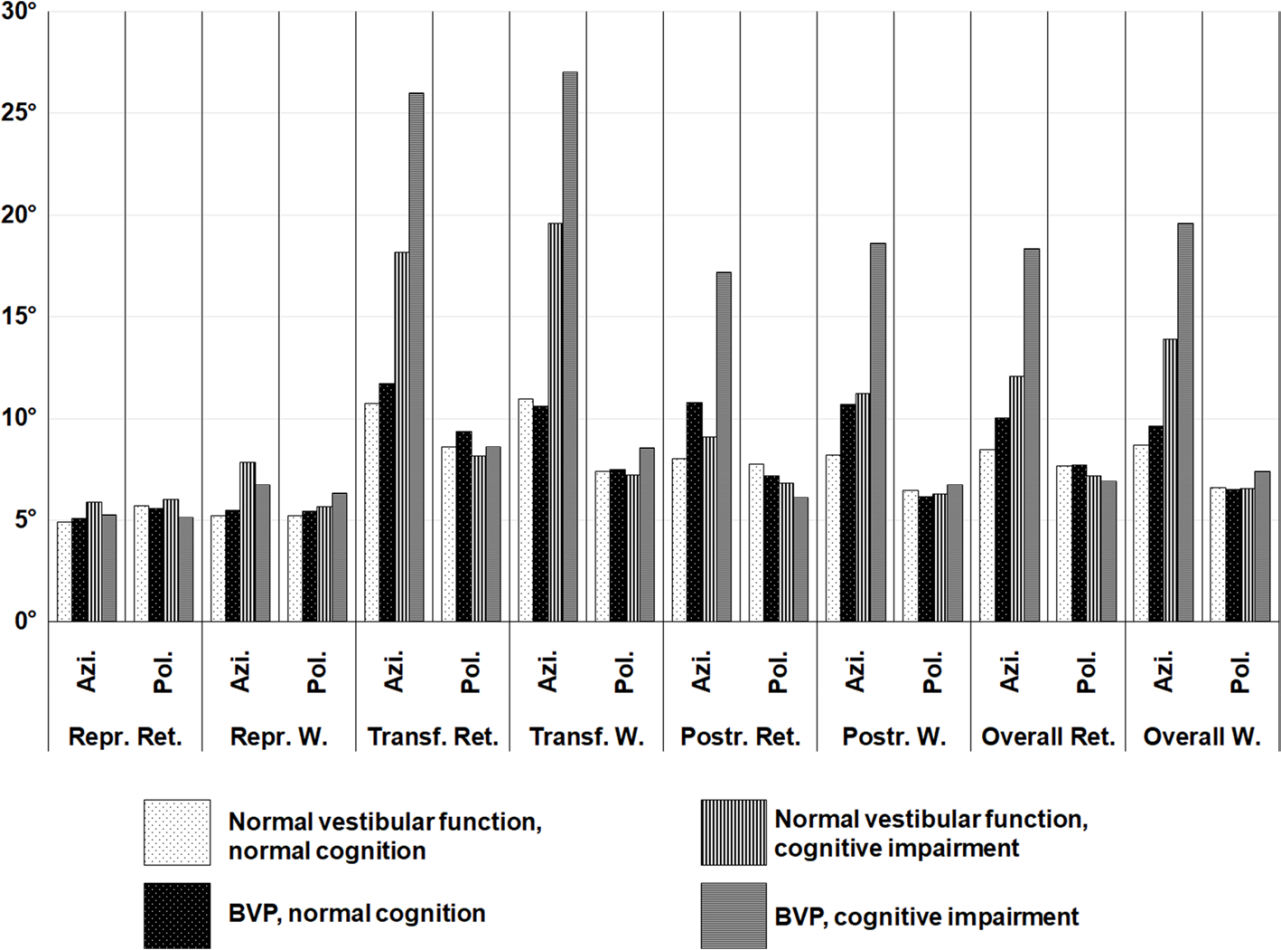
Mean angular deviations per paradigm and calibration (retinotopic, world-based) in degrees (°), displayed separated in azimuth (A) and polar (P) directions. While polar (vertical) performance was mostly stable among patients and participants regardless of vestibular or cognitive impairment, patients with cognitive impairment showed higher angular deviations in the azimuth (horizontal) plane. In patients with BVP and normal cognition, only slightly increased deviations in the azimuth plane were found compared to healthy participants (HP) (white bars with black dots: participants without cognitive or vestibular impairment, HP; black bars with white dots: patients with BVP and normal cognition; vertically striped bars: patients with cognitive impairment and normal peripheral-vestibular function; horizontally lined bars: patients with cognitive impairment and BVP). Abbreviations: *Ret.* = retinotopic, *W.* = world-based, *Azi.* = azimuth, *Pol.* = polar, *Repr.* = reproduction, *Transf.* = transformation, *Postr.* = postrotation

Polar pointing performance (m*ean polar deviations, mPD)* did not show significant group differences. Detailed results of age-corrected ANCOVA-testing for mAD and mPD can be found in Additional file 1 and in Fig. 4.

**Fig. 5:**
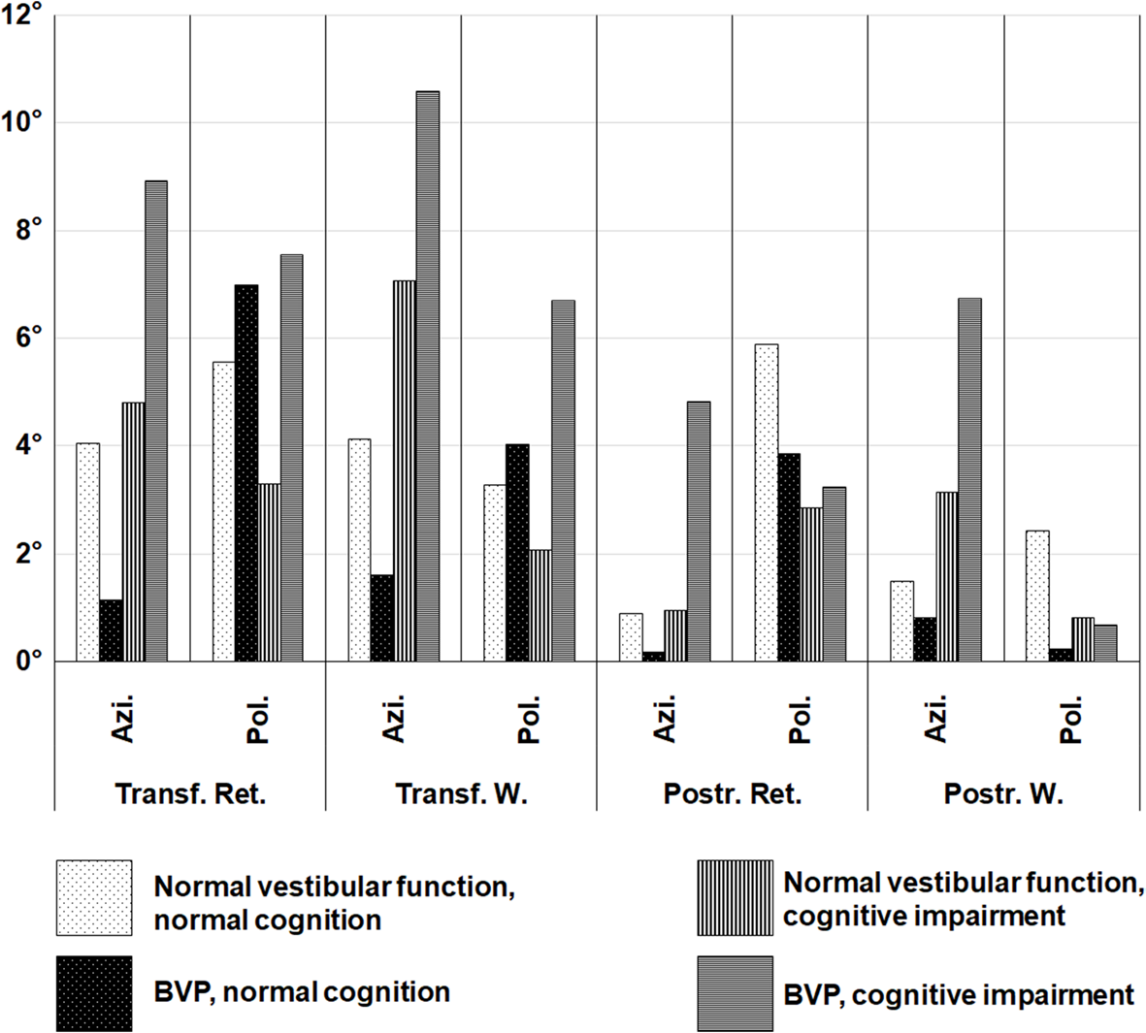
Mean directional deviation in the transformation and postrotation paradigms per calibration (retinotopic, world-based) in degrees (°). In the transformation and the postrotation paradigms BVP patients with normal cognition showed almost no directional deviation in the azimuth direction (i.e., the plane of the stimulus), while patients with an additional cognitive impairment showed large deviations, suggesting a cognitive compensation mechanism of vestibular hypofunction (white bars with black dots: participants without cognitive or vestibular impairment; black bars with white dots: patients with BVP and normal cognition; vertically striped bars: patients with cognitive impairment and normal peripheral-vestibular function; horizontally lined bars: patients with cognitive impairment and BVP)

*Mean directional deviations* (mean azimuth directional deviation, mAdD; mean polar directional deviation, mPdD), i.e., the “precision”-marker, showed a tendency towards higher (= worse) values in cognitively impaired patients, again only in the azimuth direction. Without reaching statistical significance in the age-corrected ANCOVA due to high interindividual variance, a trend was seen where this “precision” was highest in the BVP subgroup with normal cognition and lowest in BVP patients with additional cognitive impairment while the other subgroups lay in between (Fig. 5). In the analysis of paradigm-wise standard deviations as a marker for participant consistency (mean azimuth pointing consistency, mAPC, and mean polar pointing consistency, mPPC), higher values indicating a larger variance of angular deviations and higher mean standard deviations were observable in both cognitive and vestibular hypofunction. Detailed results for directional deviation and paradigm-wise consistency can be found in Additional file 1.

MoCA scores correlated with azimuth performance (Spearman’s rho: MoCA/mAD_retinotopic_ -0.53, *p* < 0.001***; MoCA/mAD_world−based_ -0.52, *p* < 0.001***) but not with polar performance (Spearman’s rho: MoCA/mPD_retinotopic_ 0.02, p 0.85; MoCA/mPD_world−based_ -0.14, p 0.16). Similarly, both directional deviations as well as overall consistency were primarily affected in the azimuth plane (Additional file 1). No direct correlation between MOCA-scores and the numerical value of preferred pointing strategy was found (Spearman’s rho: MOCA/Azimuth pointing strategy 0.14, p 0.15; MOCA/Polar pointing strategy 0.15, p 0.12).

**Fig. 6:**
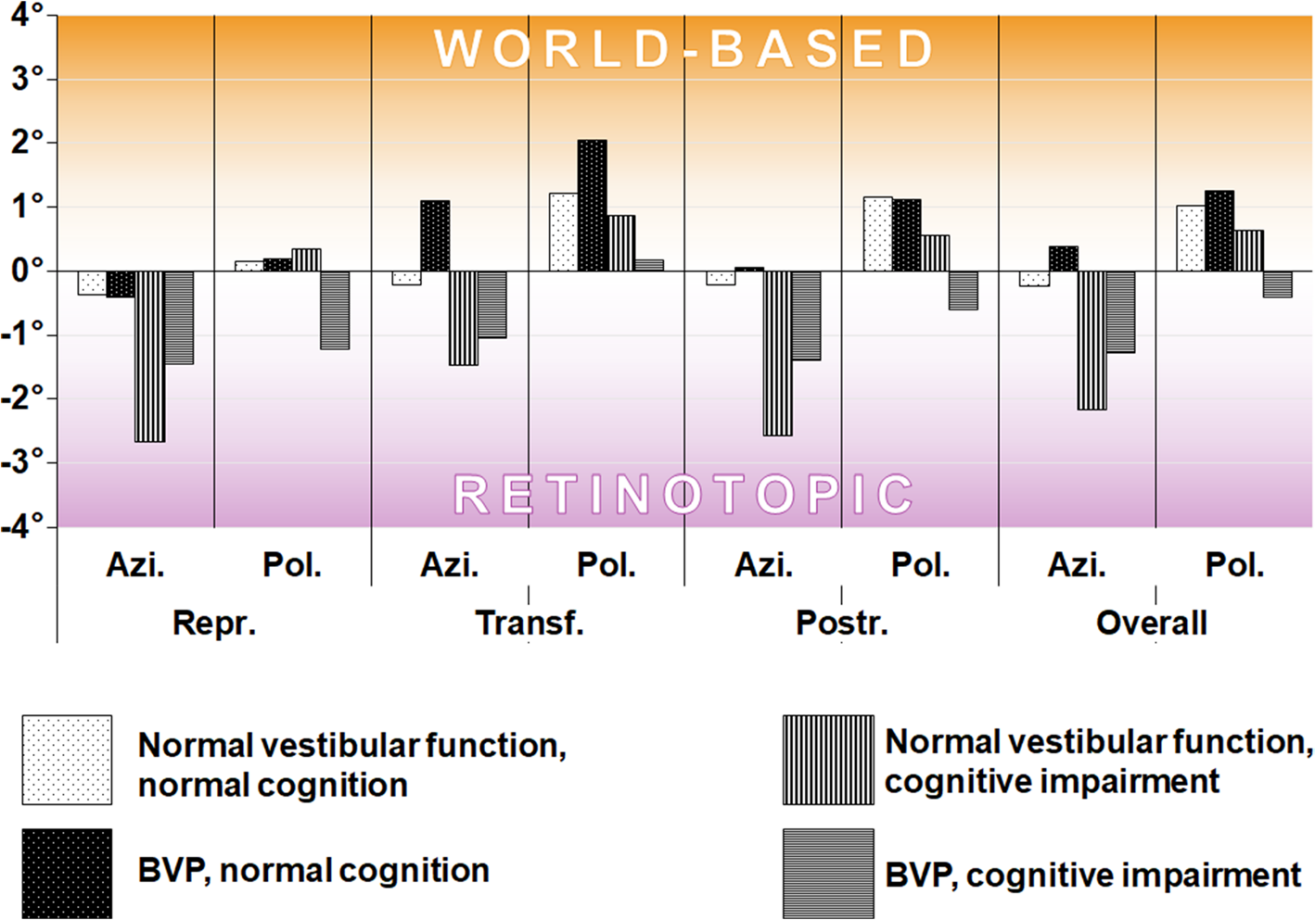
Mean difference between deviation from world-based and retinotopic calibration in degrees (°). The preferred pointing strategy can be derived by subtracting the world-based deviation from the retinotopic deviation. A subject using mostly retinotopic spatial encoding will exhibit higher angular deviations in the world-based measurement, resulting in a negative value. This can be observed in azimuth direction in participants with cognitive impairment, while participants with normal cognition tend towards a world-based pattern (white bars with black dots: participants without cognitive or vestibular impairment, HP; black bars with white dots: patients with BVP and normal cognition; vertically striped bars: patients with cognitive impairment and normal peripheral-vestibular function; horizontally lined bars: patients with cognitive impairment and BVP)

To determine the employed pointing strategy, we assessed if patients showed lower paradigm-wise deviations from their retinotopic calibration or from their world-based-calibration, respectively. Here, a-priori group differences in the respective calibration steps needed to be ruled out. For this, we performed an independent one-way ANCOVA of the groupwise mean subject-specific offset between retinotopic and world-based calibration in the azimuth and polar planes. This revealed no significant group differences in regard to cognitive and peripheral-vestibular function on mean azimuth or polar deviation (Additional file 1), therefore allowing analysis of the employed pointing strategy. When assessing the deviation difference between retinotopic and world-based spatial coding strategy, a significant shift towards the retinotopic calibration was found in transformation and post-rotation paradigms as well as subsequently in the overall analysis in patients with cognitive impairment, but only in the azimuth direction (ANCOVA normal peripheral-vestibular function vs. BVP: mean overall azimuth pointing strategy difference − 0.58, pBonf 0.32, mean overall polar pointing strategy difference 0.50, pBonf 0.25; normal cognition vs. cognitive impairment: mean overall azimuth pointing strategy difference 1.72, pBonf 5.78 × 10-3**, mean overall polar difference 0.83, pBonf 0.07). No additional effect of vestibular function was seen: both HP and BVP-patients with normal cognition tended towards world-based encoding strategies (Fig. 6).

An *egocentric fallback* pattern (i.e., a 90° degree shifted pointing pattern in the transformation tasks, as if no rotation had taken place [23]) in the rotational tasks was observed in 8 patients, but in none of the healthy participants: 5 patients with cognitive impairment and normal vestibular function, 2 patients with BVP and cognitive impairment, and one patient with BVP and normal cognition. The patients showing this pattern had significantly lower MoCA-scores both in comparison with all other participants (independent samples student’s t-test: mean difference MoCA-scores 5.61, t 4.81, *p* < 0.001***, Cohen’s d 1.77), and in comparison to the other cognitively impaired patients (independent samples student’s t-test: mean difference MOCA-scores 2.66, t 2.12, p 0.04*, Cohen’s d 0.44).

*Figure frame analysis* revealed a tendency towards increased figure area and figure perimeter in both cognitive and vestibular impairment in the reproduction paradigm (ANCOVA normal peripheral-vestibular function vs. BVP: mean reproduction area difference − 27319.84, pBonf 0.02*, Cohen’s d -0.54; normal cognition vs. cognitive impairment: mean reproduction area difference − 23947.28, pBonf 0.05*, Cohen’s d -0.47). In the transformation tasks (and subsequently the overall analysis), post-hoc testing showed increased figure frame areas in cognitive impairment, whereas BVP was not a statistically significant discriminatory factor (ANCOVA, normal cognition vs. cognitive impairment: overall area difference pBonf 0.02*, detailed results in Additional file 1).

Morphometrical figure frame analysis in transformation and postrotation paradigms showed increased perimeters and maximum feret diameters in the cognitively impaired cohort but no significant differences in regard to vestibular function (ANCOVA, normal cognition vs. cognitive impairment: max. Feret diameter difference: transformation pBonf 1.51 × 10-3**, postrotation: pBonf 0.04*; perimeter difference transformation: pBonf < 0.001***, postrotation: pBonf 0.01**, detailed results in Additional file 1). A groupwise plot of the resulting figure frames illustrates these disturbed spatial representations (Fig. 2). A directional horizontal offset following the passive whole-body rotation without visual cues was partially seen in the BVP group, while the group with cognitive impairment did not show systematic shifts (detailed ANCOVA-results in Additional file 1) The vertical center was not affected by the rotation.

## Discussion

Patients with cognitive impairment and BVP showed reduced accuracy of spatial orientation and memory in a 3D-real-world finger pointing task in a distinct manner: patients with cognitive impairment struggled in the transformation tasks (i.e., 90° rotation to the side with visual feedback available), and patients with vestibular hypofunction struggled in the post-rotation tasks (i.e., rotation back into forward-facing position; rotation performed without visual feedback available). Further, the 3D-RWPT revealed a shift of pointing strategy from world-based/allocentric towards retinotopic/egocentric pointing in patients with cognitive impairment. Individual figure frame analyses of the eight border targets of the stimulus array showed morphological alterations in the cognitively impaired patients, which were especially pronounced in the group with a combination of vestibular and cognitive deficits. This supports the view that the cognitive deficits hindered the mental buildup of the stimulus pattern represented as a geometrical form, potentially as a mild form of simultanagnosia.

A decrease in spatial orientation and navigation has been previously described for unilateral and bilateral vestibulopathy in other virtual and real-world spatial orientation and navigation tests [8, 9, 41–43]. The same is true for patients with mild cognitive impairment and dementia [7, 44, 45]. In the current study, BVP patients with an additional cognitive deficit showed severe impairment of spatial orientation, a finding well in line with another study describing an association between vestibular loss and cognitive impairment using conditional logistic regression models [14]. In our study, patients with BVP without cognitive impairment, however, in some cases showed a similarly precise directional performance as healthy participants. The latter finding has also been discussed in a recent review on the interaction between the vestibular system, memory, hippocampus, and the striatum [8]. For the postrotation tasks in our study, i.e., paradigms that rely on vestibular function [46, 47], BVP patients with normal cognition showed almost no *directional* deviation on a group level while still exhibiting increased *absolute* deviations, hinting at an intact mental representation of the targets. A mostly normal performance was observed in the transformation tasks in BVP patients with normal cognition (Fig. 5). As discussed earlier [48], one possible explanation could be a cognitive compensatory mechanism of the rotation perception, because participants were informed that the rotation without visual feedback would put them back into the initial position, facing the target matrix again straight ahead. This would be in agreement with some BVP patients’ self-report of unaffected spatial performance despite their objectively proven vestibular loss. An additional cognitive deficit, however, might impede this cognitive compensation. The finding that a “compensation” was only seen in azimuth direction is in line with orientation and locomotion preferably optimized in humans and other ground-based species in the horizontal plane [49, 50]. This might also explain why the self-assessment-questionnaire of orientation skills [15] investigated in our study cohort corresponded more with azimuth performance than polar performance (Fig. 3). Another possible confounding factor might be due to a general spatial memory distortion of 3D structures which is biased towards equilateral shapes of height and width: “taller and shorter” [51, 52].

In the current study, a shift towards egocentric mental representation in a heterogeneous group of patients with cognitive impairment could be seen as evident in the preferred pointing strategy applied. An impaired world-based (allocentric) performance with an associated shift to egocentric navigation strategies has been observed in different forms of dementia using navigation tasks, showing clear bias towards more egocentric navigation patterns in patients with, e.g., Alzheimer’s dementia [53–55]. Old age in general seems to impair switching towards allocentric navigation strategies [56]. For vestibular dysfunction, one recent study showed no clear tendency towards allo- or egocentric navigation strategies in a virtual environment [57]. The authors of the latter study explained this finding partially with the time course of vestibular hypofunction in their patient cohort. Thus, our study appears to be the first to demonstrate a shift towards retinotopic/egocentric spatial strategies in patients with cognitive decline, independent of the particular diagnosis.

In our patient cohort, the 3D-RWPT performance could discriminate between cognitive impairment and normal cognition, especially when considering both angular deviation and the employed pointing strategy. In participants with normal cognitive function, it could not reliably discriminate BVP patients from healthy participants. However, this was not the aim of our study, since other reliable diagnostic criteria for the diagnosis of BVP exist [26]. The clinical relevance of performing 3D RWPT in patients with BVP is to disclose a combination of vestibular loss and impending dementia because both conditions are not rare in the elderly. If a combination is diagnosed, cognitive training including spatial orientation and navigation can be beneficial. The latter also includes the long-term evaluation of visuospatial deficits following an acute unilateral vestibular loss [58].

A relevant limitation of this study lies in the heterogeneity of the cognitive impairment subgroup which included a variety of different etiologies and duration and stage of disease. Here, further research with more homogenous groups of e.g., solely Alzheimer’s dementia, and more detailed clinical data including A/T/N-classification and disease duration, would be desirable. A second limitation is the rather small overlap group of vestibular and cognitive deficits (*n* = 9). For future research, this group should be expanded. Furthermore, collecting 3D-RWPT data in otherwise healthy elderly adults in order to define age-specific cutoffs of expected angular accuracy, could improve the diagnostic value of the test.

In conclusion, the 3D-RWPT offers a fast way of measuring real-world spatial performance and the underlying spatial encoding strategy. It does not require complex setups or a lot of participant instruction, given that it utilizes a common everyday sensorimotor task (pointing). While the accuracy and kinematics in stationary three-dimensional subject-world interaction tasks such as pointing or grasping have been examined in a variety of neurological conditions such as movement disorders or autism [59–61], the individual method of interaction (i.e., underlying spatial coding and employed strategy) is often neglected. Compared to other potential biophysical biomarkers of spatial abilities (such as virtual reality (VR) testing setups which methodically elicit a discrepancy of visual and vestibular input [62]), it provides a physiological stimulus in a real-world environment. It offers additional insight compared to questionnaires or pen-and-paper tests, while not requiring complex setups like real-world-navigation tests. As discussed above, it appears that a combination of parameters (such as employed spatial encoding strategy, overall precision, overall accuracy, resulting shape configuration) is required to adequately assess spatial performance and uncover disease-specific subtypes of impairment of spatial orientation. It might be suitable to disclose disease specific differences in other conditions such as Parkinson’s disease, Lewy body dementia, frontotemporal dementia or posterior cortical atrophy. Pointing at remembered targets with additional transformation paradigms can therefore add valuable insights not only into preclinical stages of cognitive decline but also into the vestibular aspects of spatial cognition. Further research is needed especially in longitudinal patient cohorts with clearly defined cognitive deficits to assess the clinical significance of impaired accuracy/precision or strategy shift in the 3D-RWPT as a potential biomarker.

### Supplementary Information

**Supplementary Material 1.**

## Data Availability

The data that support the findings of this study are not publicly available due to patient and participant privacy, but anonymized group- or paradigm-wise datasets are available on reasonable request from the corresponding author [JG].
