# Supplementary material for: A clinical 3D pointing test differentiates spatial memory deficits in dementia and bilateral vestibular failure
Source: BMC Neurol. 2024 Feb 23;24:75. doi: 10.1186/s12883-024-03569-4 (PMC10885646; doi:10.1186/s12883-024-03569-4)
Supplement: Supplementary file 1 — Supplementary Material 1. [file 12883_2024_3569_MOESM1_ESM.docx]

**Additional file 1**

# 1

SBSODS: emotional, functional, neutral subsets and overall score

ANCOVA (corrected for age):

- mean difference **emotional** subset
  - normal peripheral-vestibular function vs. BVP: F(1,106)=1.39, p 0.24, partial η2=0.01, post-hoc difference = -0.32, pBonf 0.24;
  - normal cognition vs. cognitive impairment: F(1,106)=0.52, p 0.47, partial η2=4.72x10-3, post-hoc difference = 0.21, pBonf 0.47;
- mean difference **functional** subset
  - normal peripheral-vestibular function vs. BVP: F(1,106)=0.15, p 0.70, partial η2=1.43x10-3, post-hoc difference = -0.10, pBonf 0.70;
  - normal cognition vs. cognitive impairment: F(1,106)=0.13, p 0.72, partial η2=1.21x10-3, post-hoc difference = 0.09, pBonf 0.72;
- mean difference **neutral** subset
  - normal peripheral-vestibular function vs. BVP: F(1,106)=0.75, p 0.39, partial η2=6.40x10-3, post-hoc difference = -0.27, pBonf 0.39;
  - normal cognition vs. cognitive impairment: F(1,106)=0.27, p 0.60, partial η2=2.34x10-3, post-hoc difference = 0.17, pBonf 0.60;
- mean difference **overall** score
  - normal peripheral-vestibular function vs. BVP: F(1,106)=0.59, p 0.45, partial η2=5.46x10-3, post-hoc difference =–0.17, pBonf 0.45;
  - normal cognition vs. cognitive impairment: F(1,106)=0.50, p 0.48, partial η2=4.67x10-3, post-hoc difference = 0.17, pBonf 0.48.

# 2

Mean overall Azimuth deviations

ANCOVA (corrected for age):

- retinotopic: normal peripheral-vestibular function vs. BVP: mean difference -3.87°, pBonf <0.001***, Cohen’s d -0.84, normal cognition vs. cognitive impairment: mean difference -5.18°, pBonf <0.001***, Cohen’s d -1.12;
- world-based: normal peripheral-vestibular function vs. BVP: mean difference -3.29°, pBonf 3.75x10-3**, Cohen’s d -0.65, normal cognition vs. cognitive impairment: mean difference -6.90°, pBonf <0.001***, Cohen’s d -1.36)

# 3

Mean azimuth deviations (mAD) and mean polar deviations (mPD) and results of ANCOVA (corrected for age) testing of groupwise differences.

|  | Paradigm | Calibration | Post-Hoc: normal vestibular function vs. BVP: mean difference | Post-Hoc: normal vestibular function vs. BVP: pBonf | Cohen's d | Post-Hoc: normal cognition vs. cognitive impairment: mean difference | Post-Hoc: normal cognition vs. cognitive impairment: mean difference: pBonf | Cohen's d |  | Mean | SD |
| --- | --- | --- | --- | --- | --- | --- | --- | --- | --- | --- | --- |
| mAD | Reproduction | Retinotopic | 0.21 | 0.73 | - | -0.37 | 0.57 | - | BVP, normal cognition | 5.09 | 3.03 |
|  |  |  |  |  |  |  |  |  | BVP, cognitive impairment | 5.27 | 1.38 |
|  |  |  |  |  |  |  |  |  | normal vestibular function, normal cognition | 4.90 | 2.76 |
|  |  |  |  |  |  |  |  |  | normal vestibular function, cognitive impairment | 5.87 | 2.63 |
|  |  | World-based | 0.43 | 0.61 | - | **-1.83** | **0.04*** | **-0.48** | BVP, normal cognition | 5.50 | 4.13 |
|  |  |  |  |  |  |  |  |  | BVP, cognitive impairment | 6.72 | 1.71 |
|  |  |  |  |  |  |  |  |  | normal vestibular function, normal cognition | 5.23 | 2.84 |
|  |  |  |  |  |  |  |  |  | normal vestibular function, cognitive impairment | 7.85 | 5.62 |
|  | Transformation | Retinotopic | **-4.36** | **0.02*** | **-0.53** | **-9.64** | **<0.001***** | **-1.18** | BVP, normal cognition | 11.71 | 5.46 |
|  |  |  |  |  |  |  |  |  | BVP, cognitive impairment | 25.99 | 17.34 |
|  |  |  |  |  |  |  |  |  | normal vestibular function, normal cognition | 10.75 | 5.38 |
|  |  |  |  |  |  |  |  |  | normal vestibular function, cognitive impairment | 18.15 | 11.80 |
|  |  | World-based | **-3.52** | **0.05*** | **-0.44** | **-11.51** | **<0.001***** | **-1.42** | BVP, normal cognition | 10.62 | 3.84 |
|  |  |  |  |  |  |  |  |  | BVP, cognitive impairment | 27.04 | 16.68 |
|  |  |  |  |  |  |  |  |  | normal vestibular function, normal cognition | 10.95 | 5.04 |
|  |  |  |  |  |  |  |  |  | normal vestibular function, cognitive impairment | 19.60 | 12.69 |
|  | Postrotation | Retinotopic | **-5.43** | **<0.001***** | **-1.13** | **-3.12** | **6.17x10-3**** | **-0.65** | BVP, normal cognition | 10.78 | 5.53 |
|  |  |  |  |  |  |  |  |  | BVP, cognitive impairment | 17.20 | 10.47 |
|  |  |  |  |  |  |  |  |  | normal vestibular function, normal cognition | 8.01 | 3.63 |
|  |  |  |  |  |  |  |  |  | normal vestibular function, cognitive impairment | 9.07 | 3.79 |
|  |  | World-based | **-4.93** | **<0.001***** | **-0.86** | **-4.83** | **<0.001***** | **-0.84** | BVP, normal cognition | 10.71 | 6.81 |
|  |  |  |  |  |  |  |  |  | BVP, cognitive impairment | 18.60 | 10.98 |
|  |  |  |  |  |  |  |  |  | normal vestibular function, normal cognition | 8.20 | 3.45 |
|  |  |  |  |  |  |  |  |  | normal vestibular function, cognitive impairment | 11.21 | 6.51 |
|  | Overall | Retinotopic | **-3.87** | **<0.001***** | **-0.84** | **-5.18** | **<0.001***** | **-1.12** | BVP, normal cognition | 10.01 | 4.32 |
|  |  |  |  |  |  |  |  |  | BVP, cognitive impairment | 18.33 | 9.71 |
|  |  |  |  |  |  |  |  |  | normal vestibular function, normal cognition | 8.49 | 3.53 |
|  |  |  |  |  |  |  |  |  | normal vestibular function, cognitive impairment | 12.06 | 5.21 |
|  |  | World-based | **-3.29** | **3.75x10-3**** | **-0.65** | **-6.90** | **<0.001***** | **-1.36** | BVP, normal cognition | 9.63 | 4.51 |
|  |  |  |  |  |  |  |  |  | BVP, cognitive impairment | 19.60 | 9.50 |
|  |  |  |  |  |  |  |  |  | normal vestibular function, normal cognition | 8.71 | 3.23 |
|  |  |  |  |  |  |  |  |  | normal vestibular function, cognitive impairment | 13.89 | 7.18 |
| mPD | Reproduction | Retinotopic | 0.53 | 0.36 | - | 0.21 | 0.73 | - | BVP, normal cognition | 5.58 | 1.81 |
|  |  |  |  |  |  |  |  |  | BVP, cognitive impairment | 5.11 | 2.37 |
|  |  |  |  |  |  |  |  |  | normal vestibular function, normal cognition | 5.73 | 3.00 |
|  |  |  |  |  |  |  |  |  | normal vestibular function, cognitive impairment | 6.01 | 2.52 |
|  |  | World-based | -0.45 | 0.43 | - | -0.50 | 0.40 | - | BVP, normal cognition | 5.43 | 1.72 |
|  |  |  |  |  |  |  |  |  | BVP, cognitive impairment | 6.34 | 2.90 |
|  |  |  |  |  |  |  |  |  | normal vestibular function, normal cognition | 5.21 | 2.89 |
|  |  |  |  |  |  |  |  |  | normal vestibular function, cognitive impairment | 5.66 | 2.51 |
|  | Transformation | Retinotopic | -0.62 | 0.35 | - | 0.61 | 0.38 | - | BVP, normal cognition | 9.35 | 2.76 |
|  |  |  |  |  |  |  |  |  | BVP, cognitive impairment | 8.61 | 3.15 |
|  |  |  |  |  |  |  |  |  | normal vestibular function, normal cognition | 8.58 | 2.93 |
|  |  |  |  |  |  |  |  |  | normal vestibular function, cognitive impairment | 8.15 | 3.13 |
|  |  | World-based | -0.70 | 0.20 | - | -0.21 | 0.72 | - | BVP, normal cognition | 7.48 | 2.27 |
|  |  |  |  |  |  |  |  |  | BVP, cognitive impairment | 8.55 | 3.32 |
|  |  |  |  |  |  |  |  |  | normal vestibular function, normal cognition | 7.39 | 2.36 |
|  |  |  |  |  |  |  |  |  | normal peripheral-vestibular function, cognitive impairment | 7.23 | 2.69 |
|  | Postrotation | Retinotopic | 0.64 | 0.37 | - | 1.05 | 0.16 | - | BVP, normal cognition | 7.18 | 3.10 |
|  |  |  |  |  |  |  |  |  | BVP, cognitive impairment | 6.13 | 2.18 |
|  |  |  |  |  |  |  |  |  | normal vestibular function, normal cognition | 7.76 | 3.12 |
|  |  |  |  |  |  |  |  |  | normal vestibular function, cognitive impairment | 6.81 | 3.78 |
|  |  | World-based | -0.05 | 0.94 | - | -0.16 | 0.82 | - | BVP, normal cognition | 6.14 | 2.54 |
|  |  |  |  |  |  |  |  |  | BVP, cognitive impairment | 6.73 | 2.45 |
|  |  |  |  |  |  |  |  |  | normal vestibular function, normal cognition | 6.46 | 2.69 |
|  |  |  |  |  |  |  |  |  | normal vestibular function, cognitive impairment | 6.28 | 2.83 |
|  | Overall | Retinotopic | 0.11 | 0.84 | - | 0.71 | 0.25 | - | BVP, normal cognition | 7.73 | 2.07 |
|  |  |  |  |  |  |  |  |  | BVP, cognitive impairment | 6.92 | 2.30 |
|  |  |  |  |  |  |  |  |  | normal vestibular function, normal cognition | 7.68 | 2.71 |
|  |  |  |  |  |  |  |  |  | normal vestibular function, cognitive impairment | 7.19 | 2.98 |
|  |  | World-based | -0.39 | 0.42 | - | -0.13 | 0.84 | - | BVP, normal cognition | 6.53 | 1.72 |
|  |  |  |  |  |  |  |  |  | BVP, cognitive impairment | 7.38 | 2.33 |
|  |  |  |  |  |  |  |  |  | normal vestibular function, normal cognition | 6.58 | 2.28 |
|  |  |  |  |  |  |  |  |  | normal vestibular function, cognitive impairment | 6.54 | 2.33 |

# 4

Mean Azimuth deviations in the transformation task

ANCOVA (corrected for age):

- retinotopic: normal peripheral-vestibular function vs. BVP: mean difference -4.36°, pBonf 0.02*, Cohen’s d -0.53, normal cognition vs. cognitive impairment: mean difference -9.64°, pBonf <0.001***, Cohen’s d -1.18;
- world-based: normal peripheral-vestibular function vs. BVP: mean difference -3.52°, pBonf 0.05*, Cohen’s d -0.44, normal cognition vs. cognitive impairment: mean difference -11.51°, pBonf <0.001***, Cohen’s d -1.42

# 5

Mean Azimuth deviations in the post-rotation task

ANCOVA (corrected for age):

- retinotopic: normal peripheral-vestibular function vs. BVP: mean difference -5.43°, pBonf <0.001***, Cohen’s d -1.13, normal cognition vs. cognitive impairment: mean difference -3.12°, pBonf 6.17x10-3**, Cohen’s d -0.65;
- world-based: normal peripheral-vestibular function vs. BVP: mean difference -4.93°, pBonf <0.001***, Cohen’s d -0.86, normal cognition vs. cognitive impairment: mean difference -4.83°, pBonf <0.001***, Cohen’s d -0.84)

# 6

Mean azimuth directional deviations (mAdD) and mean polar directional deviations (mPdD). Given that unsystematic performance might average out to low directional deviations, we also provide metrics for participant consistency derived from the paradigm-wise standard deviations (mean azimuth pointing consistency, mAPC, and mean polar pointing consistency, mPPC).

|  | Paradigm | Calibration | Post-Hoc: normal vestibular function vs. BVP: mean difference | Post-Hoc: normal vestibular function vs. BVP: pBonf | Cohen's d | Post-Hoc: normal cognition vs. cognitive impairment: mean difference | Post-Hoc: normal cognition vs. cognitive impairment: mean difference: pBonf | Cohen's d |  | Mean | SD |
| --- | --- | --- | --- | --- | --- | --- | --- | --- | --- | --- | --- |
| mAdD | Reproduction | Retinotopic | -1.42 | 0.15 | - | -0.85 | 0.41 | - | BVP, normal cognition | 1.06 | 4.07 |
|  |  |  |  |  |  |  |  |  | BVP, cognitive impairment | 3.24 | 2.81 |
|  |  |  |  |  |  |  |  |  | normal vestibular function, normal cognition | 1.12 | 4.62 |
|  |  |  |  |  |  |  |  |  | normal vestibular function, cognitive impairment | 0.36 | 4.71 |
|  |  | World-based | -1.29 | 0.30 | - | -2.38 | 0.07 | - | BVP, normal cognition | 1.69 | 6.02 |
|  |  |  |  |  |  |  |  |  | BVP, cognitive impairment | 5.15 | 3.03 |
|  |  |  |  |  |  |  |  |  | normal vestibular function, normal cognition | 1.73 | 4.60 |
|  |  |  |  |  |  |  |  |  | normal vestibular function, cognitive impairment | 2.54 | 7.50 |
|  | Transformation | Retinotopic | -0.61 | 0.70 | - | **-3.94** | **0.02*** | **-0.54** | BVP, normal cognition | 1.15 | 8.75 |
|  |  |  |  |  |  |  |  |  | BVP, cognitive impairment | 8.93 | 12.00 |
|  |  |  |  |  |  |  |  |  | normal vestibular function, normal cognition | 4.04 | 5.96 |
|  |  |  |  |  |  |  |  |  | normal vestibular function, cognitive impairment | 4.80 | 6.21 |
|  |  | World-based | -0.49 | 0.76 | - | **-5.81** | **<0.001***** | **-0.81** | BVP, normal cognition | 1.60 | 7.20 |
|  |  |  |  |  |  |  |  |  | BVP, cognitive impairment | 10.58 | 12.93 |
|  |  |  |  |  |  |  |  |  | normal vestibular function, normal cognition | 4.12 | 5.28 |
|  |  |  |  |  |  |  |  |  | normal vestibular function, cognitive impairment | 7.07 | 8.59 |
|  | Postrotation | Retinotopic | -1.58 | 0.37 | - | -2.60 | 0.14 | - | BVP, normal cognition | 0.18 | 8.33 |
|  |  |  |  |  |  |  |  |  | BVP, cognitive impairment | 4.82 | 18.66 |
|  |  |  |  |  |  |  |  |  | normal vestibular function, normal cognition | 0.90 | 5.88 |
|  |  |  |  |  |  |  |  |  | normal vestibular function, cognitive impairment | 0.96 | 6.27 |
|  |  | World-based | -1.46 | 0.47 | - | **-4.14** | **0.05*** | **-0.46** | BVP, normal cognition | 0.81 | 9.44 |
|  |  |  |  |  |  |  |  |  | BVP, cognitive impairment | 6.73 | 19.78 |
|  |  |  |  |  |  |  |  |  | normal vestibular function, normal cognition | 1.50 | 5.67 |
|  |  |  |  |  |  |  |  |  | normal vestibular function, cognitive impairment | 3.14 | 9.56 |
|  | Overall | Retinotopic | -1.74 | 0.21 | - | **-3.00** | **0.04*** | **-0.48** | BVP, normal cognition | 0.86 | 6.34 |
|  |  |  |  |  |  |  |  |  | BVP, cognitive impairment | 6.42 | 12.92 |
|  |  |  |  |  |  |  |  |  | normal vestibular function, normal cognition | 1.85 | 5.06 |
|  |  |  |  |  |  |  |  |  | normal vestibular function, cognitive impairment | 1.95 | 5.50 |
|  |  | World-based | -1.61 | 0.29 | - | **-4.54** | **5.80x10-3**** | **-0.65** | BVP, normal cognition | 1.49 | 6.48 |
|  |  |  |  |  |  |  |  |  | BVP, cognitive impairment | 8.32 | 13.81 |
|  |  |  |  |  |  |  |  |  | normal vestibular function, normal cognition | 2.46 | 4.86 |
|  |  |  |  |  |  |  |  |  | normal vestibular function, cognitive impairment | 4.15 | 8.41 |
| mPdD | Reproduction | Retinotopic | 0.30 | 0.73 | - | 1.33 | 0.15 | - | BVP, normal cognition | 2.11 | 4.00 |
|  |  |  |  |  |  |  |  |  | BVP, cognitive impairment | 2.03 | 4.81 |
|  |  |  |  |  |  |  |  |  | normal vestibular function, normal cognition | 3.38 | 3.81 |
|  |  |  |  |  |  |  |  |  | normal vestibular function, cognitive impairment | 1.35 | 3.93 |
|  |  | World-based | 0.76 | 0.42 | - | 0.47 | 0.64 | - | BVP, normal cognition | -1.51 | 4.09 |
|  |  |  |  |  |  |  |  |  | BVP, cognitive impairment | -0.72 | 6.77 |
|  |  |  |  |  |  |  |  |  | normal vestibular function, normal cognition | -0.07 | 3.99 |
|  |  |  |  |  |  |  |  |  | normal vestibular function, cognitive impairment | -0.68 | 4.36 |
|  | Transformation | Retinotopic | **-2.84** | **0.03*** | **-0.49** | 0.87 | 0.52 | - | BVP, normal cognition | 6.99 | 4.25 |
|  |  |  |  |  |  |  |  |  | BVP, cognitive impairment | 7.55 | 8.93 |
|  |  |  |  |  |  |  |  |  | normal vestibular function, normal cognition | 5.56 | 5.26 |
|  |  |  |  |  |  |  |  |  | normal vestibular function, cognitive impairment | 3.30 | 7.33 |
|  |  | World-based | **-2.67** | **0.03*** | **-0.49** | -0.31 | 0.81 | - | BVP, normal cognition | 4.02 | 4.70 |
|  |  |  |  |  |  |  |  |  | BVP, cognitive impairment | 6.69 | 10.38 |
|  |  |  |  |  |  |  |  |  | normal vestibular function, normal cognition | 3.27 | 4.57 |
|  |  |  |  |  |  |  |  |  | normal vestibular function, cognitive impairment | 2.08 | 5.92 |
|  | Postrotation | Retinotopic | 0.81 | 0.43 | - | 1.77 | 0.11 | - | BVP, normal cognition | 3.86 | 5.03 |
|  |  |  |  |  |  |  |  |  | BVP, cognitive impairment | 3.24 | 3.94 |
|  |  |  |  |  |  |  |  |  | normal vestibular function, normal cognition | 5.89 | 4.46 |
|  |  |  |  |  |  |  |  |  | normal vestibular function, cognitive impairment | 2.85 | 4.94 |
|  |  | World-based | 1.17 | 0.27 | - | 0.83 | 0.46 | - | BVP, normal cognition | 0.24 | 4.98 |
|  |  |  |  |  |  |  |  |  | BVP, cognitive impairment | 0.67 | 1.99 |
|  |  |  |  |  |  |  |  |  | normal vestibular function, normal cognition | 2.42 | 4.62 |
|  |  |  |  |  |  |  |  |  | normal vestibular function, cognitive impairment | 0.81 | 4.82 |
|  | Overall | Retinotopic | -0.26 | 0.77 | - | 1.55 | 0.10 | - | BVP, normal cognition | 4.73 | 3.92 |
|  |  |  |  |  |  |  |  |  | BVP, cognitive impairment | 4.19 | 3.94 |
|  |  |  |  |  |  |  |  |  | normal peripheral-vestibular function, normal cognition | 5.46 | 4.07 |
|  |  |  |  |  |  |  |  |  | normal vestibular function, cognitive impairment | 2.93 | 4.15 |
|  |  | World-based | 0.16 | 0.86 | - | 0.62 | 0.52 | - | BVP, normal cognition | 1.09 | 4.01 |
|  |  |  |  |  |  |  |  |  | BVP, cognitive impairment | 1.54 | 6.04 |
|  |  |  |  |  |  |  |  |  | normal vestibular function, normal cognition | 1.99 | 4.02 |
|  |  |  |  |  |  |  |  |  | normal vestibular function, cognitive impairment | 0.93 | 4.17 |
| mAPC | Reproduction | Retinotopic | -0.21 | 0.76 | - | 0.20 | 0.79 | - | BVP, normal cognition | 4.31 | 3.87 |
|  |  |  |  |  |  |  |  |  | BVP, cognitive impairment | 4.22 | 1.33 |
|  |  |  |  |  |  |  |  |  | normal vestibular function, normal cognition | 4.06 | 3.17 |
|  |  |  |  |  |  |  |  |  | normal vestibular function, cognitive impairment | 4.05 | 2.21 |
|  |  | World-based | 0.18 | 0.77 | - | **-1.41** | **0.03*** | **-0.51** | BVP, normal cognition | 3.73 | 2.14 |
|  |  |  |  |  |  |  |  |  | BVP, cognitive impairment | 5.42 | 1.71 |
|  |  |  |  |  |  |  |  |  | normal vestibular function, normal cognition | 4.01 | 2.75 |
|  |  |  |  |  |  |  |  |  | normal vestibular function, cognitive impairment | 5.47 | 3.50 |
|  | Transformation | Retinotopic | **-3.10** | **8.31x10-3**** | **-0.59** | **-5.78** | **<0.001***** | **-1.10** | BVP, normal cognition | 7.44 | 3.78 |
|  |  |  |  |  |  |  |  |  | BVP, cognitive impairment | 16.45 | 10.18 |
|  |  |  |  |  |  |  |  |  | normal vestibular function, normal cognition | 6.95 | 4.30 |
|  |  |  |  |  |  |  |  |  | normal vestibular function, cognitive impairment | 10.70 | 6.64 |
|  |  | World-based | **-2.67** | **0.01*** | **-0.56** | **-6.77** | **<0.001***** | **-1.41** | BVP, normal cognition | 6.61 | 2.32 |
|  |  |  |  |  |  |  |  |  | BVP, cognitive impairment | 17.09 | 9.72 |
|  |  |  |  |  |  |  |  |  | normal vestibular function, normal cognition | 7.05 | 3.68 |
|  |  |  |  |  |  |  |  |  | normal vestibular function, cognitive impairment | 11.26 | 6.77 |
|  | Postrotation | Retinotopic | -1.44 | 0.07 | - | -1.08 | 0.20 | - | BVP, normal cognition | 7.08 | 3.62 |
|  |  |  |  |  |  |  |  |  | BVP, cognitive impairment | 9.22 | 3.93 |
|  |  |  |  |  |  |  |  |  | normal vestibular function, normal cognition | 6.33 | 3.78 |
|  |  |  |  |  |  |  |  |  | normal vestibular function, cognitive impairment | 7.06 | 3.10 |
|  |  | World-based | -0.70 | 0.39 | - | **-2.41** | **6.40x10-3**** | **-0.65** | BVP, normal cognition | 6.22 | 2.48 |
|  |  |  |  |  |  |  |  |  | BVP, cognitive impairment | 9.72 | 4.99 |
|  |  |  |  |  |  |  |  |  | normal vestibular function, normal cognition | 6.30 | 3.49 |
|  |  |  |  |  |  |  |  |  | normal vestibular function, cognitive impairment | 8.21 | 4.88 |
|  | Overall | Retinotopic | **-1.85** | **0.01*** | **-0.57** | **-2.69** | **<0.001***** | **-0.83** | BVP, normal cognition | 6.67 | 3.32 |
|  |  |  |  |  |  |  |  |  | BVP, cognitive impairment | 11.12 | 4.23 |
|  |  |  |  |  |  |  |  |  | normal vestibular function, normal cognition | 6.14 | 3.06 |
|  |  |  |  |  |  |  |  |  | normal vestibular function, cognitive impairment | 7.92 | 3.31 |
|  |  | World-based | **-1.30** | **0.05*** | **-0.44** | **-3.94** | **<0.001***** | **-1.33** | BVP, normal cognition | 5.88 | 1.82 |
|  |  |  |  |  |  |  |  |  | BVP, cognitive impairment | 11.80 | 4.06 |
|  |  |  |  |  |  |  |  |  | normal vestibular function, normal cognition | 6.16 | 2.45 |
|  |  |  |  |  |  |  |  |  | normal vestibular function, cognitive impairment | 8.89 | 4.45 |
| mPPC | Reproduction | Retinotopic | 0.62 | 0.32 | - | 0.28 | 0.67 | - | BVP, normal cognition | 4.76 | 2.67 |
|  |  |  |  |  |  |  |  |  | BVP, cognitive impairment | 3.23 | 1.80 |
|  |  |  |  |  |  |  |  |  | normal vestibular function, normal cognition | 4.06 | 2.45 |
|  |  |  |  |  |  |  |  |  | normal vestibular function, cognitive impairment | 5.16 | 3.79 |
|  |  | World-based | 0.88 | 0.19 | - | -0.18 | 0.80 | - | BVP, normal cognition | 3.92 | 1.63 |
|  |  |  |  |  |  |  |  |  | BVP, cognitive impairment | 3.32 | 1.14 |
|  |  |  |  |  |  |  |  |  | normal vestibular function, normal cognition | 3.96 | 3.08 |
|  |  |  |  |  |  |  |  |  | normal vestibular function, cognitive impairment | 5.03 | 4.15 |
|  | Transformation | Retinotopic | -0.81 | 0.15 | - | -0.25 | 0.68 | - | BVP, normal cognition | 6.37 | 2.45 |
|  |  |  |  |  |  |  |  |  | BVP, cognitive impairment | 7.25 | 2.71 |
|  |  |  |  |  |  |  |  |  | normal vestibular function, normal cognition | 6.02 | 2.56 |
|  |  |  |  |  |  |  |  |  | normal vestibular function, cognitive impairment | 5.97 | 2.52 |
|  |  | World-based | -0.54 | 0.38 | - | -0.57 | 0.38 | - | BVP, normal cognition | 5.59 | 2.58 |
|  |  |  |  |  |  |  |  |  | BVP, cognitive impairment | 7.12 | 2.71 |
|  |  |  |  |  |  |  |  |  | normal vestibular function, normal cognition | 5.87 | 2.94 |
|  |  |  |  |  |  |  |  |  | normal vestibular function, cognitive impairment | 5.76 | 2.53 |
|  | Postrotation | Retinotopic | -0.26 | 0.65 | - | -0.01 | 1.00 | - | BVP, normal cognition | 5.31 | 2.83 |
|  |  |  |  |  |  |  |  |  | BVP, cognitive impairment | 5.26 | 2.69 |
|  |  |  |  |  |  |  |  |  | normal vestibular function, normal cognition | 5.00 | 2.45 |
|  |  |  |  |  |  |  |  |  | normal vestibular function, cognitive impairment | 5.05 | 2.55 |
|  |  | World-based | 0.23 | 0.69 | - | -0.25 | 0.70 | - | BVP, normal cognition | 4.66 | 2.58 |
|  |  |  |  |  |  |  |  |  | BVP, cognitive impairment | 4.88 | 2.41 |
|  |  |  |  |  |  |  |  |  | normal vestibular function, normal cognition | 4.92 | 2.83 |
|  |  |  |  |  |  |  |  |  | normal vestibular function, cognitive impairment | 5.09 | 2.50 |
|  | Overall | Retinotopic | -0.31 | 0.51 | - | -0.05 | 0.91 | - | BVP, normal cognition | 5.63 | 2.12 |
|  |  |  |  |  |  |  |  |  | BVP, cognitive impairment | 5.65 | 2.11 |
|  |  |  |  |  |  |  |  |  | normal vestibular function, normal cognition | 5.21 | 2.09 |
|  |  |  |  |  |  |  |  |  | normal vestibular function, cognitive impairment | 5.45 | 2.15 |
|  |  | World-based | 0.06 | 0.90 | - | -0.37 | 0.50 | - | BVP, normal cognition | 4.88 | 1.84 |
|  |  |  |  |  |  |  |  |  | BVP, cognitive impairment | 5.47 | 1.85 |
|  |  |  |  |  |  |  |  |  | normal vestibular function, normal cognition | 5.11 | 2.60 |
|  |  |  |  |  |  |  |  |  | normal vestibular function, cognitive impairment | 5.36 | 2.15 |

# 7

Correlation analysis of MoCA-Scores and directional deviations using Spearman’s rho

- MoCA/mAdD_retinotopic_ -0.23, p 0.02*; MoCA/mAdD_world-based_ -0.18, p 0.07;
- MoCA/mPdD_retinotopic_ 0.17, p 0.09; MoCA/mPdD_world-based_ 0.03, p 0.77;
- MoCA/mAPC_retinotopic_ -0.50, p <0.001***; MoCA/mAPC_world-based_ -0.49, p <0.001***;
- MoCA/mPPC_retinotopic_ -0.17, p 0.08; MoCA/mPPC_world-based_ -0.18, p 0.08).

# 8

Mean calibration differences, to allow for comparison of employed pointing strategy:

ANCOVA (corrected for age):

- normal peripheral-vestibular function vs. BVP:
  - azimuth F(1,130)=1.39, p 0.24, partial η^2^=9.88x10-3, post-hoc difference = 0.92°, pBonf 0.24,
  - polar F(1,130)=0.03, p 0.86, partial η^2^=2.16x10-4, post-hoc difference = -0.10°, pBonf 0.86;
- normal cognition vs. cognitive impairment:
  - azimuth F(1,130)=0.15, p 0.70, partial η^2^=1.05x10-3, post-hoc difference = 0.32°, pBonf 0.70,
  - polar F(1,130)=1.59, p 0.21, partial η^2^=0.01, post-hoc difference = 0.75°, pBonf 0.21

# 9

Mean figure frame area

ANCOVA (corrected for age):

- normal peripheral-vestibular function vs. BVP: mean transformation area difference -10887.13, pBonf 0.55; normal cognition vs. cognitive impairment: mean transformation area difference -44197.81, pBonf 0.02*, Cohen’s d -0.54;
- normal peripheral-vestibular function vs. BVP: mean postrotation area difference -17428.73, pBonf 0.27; normal cognition vs. cognitive impairment: mean postrotation area difference -28512.09, pBonf 0.09;
- normal peripheral-vestibular function vs. BVP: mean overall area difference -16790.31, pBonf 0.23; normal cognition vs. cognitive impairment: mean overall area difference -33873.52, pBonf 0.02*, Cohen’s d -0.54

# 10

Mean shape configuration differences: feret diameter, perimeter:

ANCOVA (corrected for age)

- normal peripheral-vestibular function vs. BVP: mean **transformation** max feret diameter difference -83.23, pBonf 0.08;
- normal cognition vs. cognitive impairment: mean **transformation** max feret diameter difference -163.25, pBonf 1.51x10-3**, Cohen’s d -0.76;
- normal peripheral-vestibular function vs. BVP: mean **postrotation** max feret diameter difference -54.16, pBonf 0.09;
- normal cognition vs. cognitive impairment: mean **postrotation** max feret diameter difference -70.27, pBonf 0.04*, Cohen’s d -0.48;
- normal peripheral-vestibular function vs. BVP: mean **transformation** perimeter difference -69.28, pBonf 0.05;
- normal cognition vs. cognitive impairment: mean **transformation** perimeter difference -606.35, pBonf <0.001***, Cohen’s d -0.96;
- normal peripheral-vestibular function vs. BVP: mean **postrotation** perimeter difference -132.06, pBonf 0.13;
- normal cognition vs. cognitive impairment: mean **postrotation** perimeter difference -203.25, pBonf 0.01***, Cohen’s d -0.59

# 11

Mean shape configuration differences: inertia ellipse center (x):

ANCOVA (corrected for age, world-based and retinotopic calibration inertia ellipse center x)

- mean postrotation (non-hand dominant side) center difference normal cognition/cognitive impairment 0.65, p_bonf=_0.98; center difference normal peripheral-vestibular function/bilateral deficit 48.57, p_bonf=_0.02*;
- mean postrotation (hand dominant side) center difference normal cognition/cognitive impairment 25.64, p_bonf=_0.25; center difference normal peripheral-vestibular function/bilateral deficit -27.95, p_bonf=_0.18).
